# N-acetylcysteine Ameliorates Vancomycin-induced Nephrotoxicity by Inhibiting Oxidative Stress and Apoptosis in the* in vivo* and *in vitro* Models

**DOI:** 10.7150/ijms.69807

**Published:** 2022-04-11

**Authors:** Ping Yu, Jing Luo, Huahua Song, Tianwei Qian, Xuan He, Jie Fang, Wenpei Dong, Xiaolan Bian

**Affiliations:** 1 Department of Pharmacy, Ruijin Hospital, Shanghai Jiao Tong University School of Medicine, Shanghai, China.; 2 Department of General Surgery, Huadong Hospital, Fudan University, Shanghai, China.; 3 Department of Pharmacology, Shanghai Jiao Tong University School of Medicine, Shanghai, China.

**Keywords:** vancomycin, nephrotoxicity, N-acetylcysteine, oxidative stress, renal protection

## Abstract

**Background:** Oxidative stress-related apoptosis is considered as the key mechanism implicated in the pathophysiology of nephrotoxicity with vancomycin (VCM) therapy. We evaluated the possible effects of N-acetylcysteine (NAC) on VCM-induced nephrotoxicity and the underlying mechanism.

**Methods:** VCM-induced nephrotoxicity was established using HK-2 cells and SD rats and observed by measuring cell survival, kidney histological changes, renal function and kidney injury related markers (KIM-1 and NGAL). Oxidative stress, renal cell apoptosis and the involved signaling pathways were also evaluated.

**Results:** In model rats, NAC could protect against VCM-induced acute kidney injury with histological damage, renal dysfunction, and increased Cre and BUN levels. In HK-2 cells, VCM-induced decreased cell viability was restored by NAC. In addition, increased expression of caspase-3, KIM-1 and NGAL suffering from VCM was also reversed by NAC *in vivo* and *in vitro*. NAC inhibited ROS production, decreased cell apoptosis by decreasing the Bax/Bcl-2 ratio and caspase-3 expression in HK-2 cells and regulated oxidative stress indicators in the kidney by decreasing GSH, SOD and CAT activity and increasing MDA levels. Furthermore, NAC could effectively reverse VCM-associated increased P38 MAPK/JNK phosphorylation.

**Conclusions:** The results demonstrated that NAC had a protective effect against nephrotoxicity from VCM by inhibiting oxidative stress and apoptosis *via* P38 MAPK/JNK.

## Introduction

Vancomycin (VCM) is a glycopeptide antibiotic used for serious infections caused by methicillin-resistant gram-positive bacteria, especially *Staphylococcus aureus* and *Staphylococcus epidermidis* (MRSA and MRSE, respectively) 1. However, patients treated with VCM often suffer from nephrotoxicity, which is the main adverse reaction to VCM therapy and seriously limits its clinical application 2, 3. Therefore, preventing or reducing VCM-induced nephrotoxicity has become a reality in clinical treatment. VCM-related nephrotoxicity is a complex process involving many factors and signaling pathways. Previous studies have reported that reactive oxygen species (ROS) and related metabolic events were the basis of the mechanism resulting in nephrotoxicity in VCM therapy 4, 5. Oxidative stress is defined as an imbalance between the production of free radicals and antioxidant defenses in biological systems, which leads to mitochondrial dysfunction and cell apoptosis 6, 7. In addition, oxidative stress can affect intracellular signaling pathways and their participants, such as mitotically activated protein kinases (MAPKs), which are associated with apoptosis and caspase activation in the VCM-induced acute kidney injury process 8. Given the role of oxidative stress in the pathogenesis of VCM-induced nephrotoxicity, the combination of oxidative stress with antioxidants may prevent or improve the nephrotoxicity caused by VCM therapy.

As one of the most important antioxidant defenses, N-acetylcysteine (NAC) is a free radical scavenger used to synthesize glutathione to protect against the toxic effects of several chemicals 9, 10. Extensive evidence has shown that NAC has direct and indirect antioxidant properties by increasing the concentrations of free sulfhydryl and glutathione in cells. NAC can potentially reduce the damage caused by ROS; thus, antioxidant supplementation is an effective strategy to reduce oxidative damage 11. NAC can reduce toxicity in organs such as the liver 12, heart 13 and kidney 14 by improving the antioxidant capacity and energy metabolism. Intravenous injection of NAC in rats can prevent kidney tissue damage 15. Studies have also reported that NAC had a protective effect on cisplatin induced nephrotoxicity and ototoxicity in *in vivo* and* in vitro* experiments 16, 17. Interestingly, previous studies also demonstrated that NAC could protect against acute kidney injury induced by VCM in animal models 18. A recent randomized controlled clinical trial study revealed that NAC could protect patients from VCM-induced nephrotoxicity 19. However, existing research provides a limited understanding of the potential mechanism in this process.

The aim of this study was to evaluate the protective efficacy of the antioxidant NAC in nephrotoxicity resulting from VCM treatment and the potential signal pathway. The results will further expand the clinical application of NAC.

## Materials and methods

### Cell culture

The human kidney immortalized proximal tubule epithelial cell line (HK-2) was grown in DMEM/F-12 (BIOAGRIO, USA) supplemented with 10% fetal bovine serum and 1% penicillin/streptomycin at 37 ℃ in a 5% CO_2_ atmosphere.

### Cell viability

HK-2 cells were seeded into 96-well plates (1×10^4^ cells/well) overnight. In the groups receiving combined treatment with NAC (MedChemExpress, Shanghai, China) or vitamin C (Sigma Aldrich, USA) and VCM (MedChemExpress), NAC or vitamin C was added to the cells and incubated for 2 h. Then, the cells were incubated with VCM for 24 h. Cell viability was detected using a cell counting kit (CCK-8, TargetMol, Shanghai, China) according to the manufacturer's instructions. A microplate reader was used to determine the absorbance at 450 nm (Biotech, USA).

### Western blot

HK-2 cells were lysed on ice for 20 min. Then, the protein concentration was detected using a Bradford quantitative protein detection kit (Bio-Rad, USA). Samples were subjected to 10% SDS-PAGE, and polyvinylidene fluoride membranes were incubated with one of a series of primary antibodies at 4 ℃ overnight, followed by HRP-conjugated secondary antibodies (1:2000; Beyotime, Nanjing, China) at room temperature for 1 h. The primary antibodies were as follows: caspase-3, Bax, Bcl-2, p-JNK, JNK, p-P38 MAPK, P38 MAPK (1:1000, Cell Signaling Technology); kidney injury molecule-1 (KIM-1, 1:1000, Absin, Shanghai, China); and neutrophil gelatinase-associated apolipoprotein (NGAL, 1:1000, Affinity Biosciences, USA). β-actin (1:1000, Beyotime) served as an internal control.

### Immunofluorescence staining

HK-2 cells were seeded onto glass-bottom dishes. After treatment with drugs under different conditions, the cells were fixed with 4% paraformaldehyde (Servicebio, Hubei, China). Then, the cells were incubated with 1% bovine serum albumin for 60 min at room temperature. The samples were then incubated with caspase-3 at 4 °C for more than 12 h, followed by Alexa Fluor 488 fluorescently labeled secondary antibodies for 1 h at room temperature in the dark. Finally, 4',6-diamidino-2-phenylindole (DAPI, 1:5000, Beyotime) was added to stain the nuclei. The protein expression was observed under a laser confocal microscope.

### ROS detection

Intracellular ROS production was measured by the ROS‐specific fluorescent dye DCFH‐DA according to the manufacturer's protocol (Beyotime). HK-2 cells were seeded into 96-well plates (1×10^4^ cells/well) overnight. After treatment with drugs under different conditions, the cells were washed twice with PBS and incubated with DCFH‐DA (1:1000) for 20 min at 37 °C. After three washes with serum-free medium, the fluorescence intensity was measured by a fluorescence microplate reader. Then, images were captured by fluorescence microscopy.

### Rat kidney injury model induced by VCM

Male Sprague-Dawley (SD) rats (200-250 g) were maintained in a standard environment with 30-55% humidity at 22-25 ℃ in a 12-hr light:dark cycle. The rats were provided with ad libitum food and tap water. All animals were randomly assigned to 8 groups according to random number table, and each group consisted of five rats. There was a blinded evaluation of the results while the analysts do not know the exact groups of animals. Experiments were performed according to the principles and guidelines of the National Institutes of Health Guide for the Care and Use of Laboratory Animals. Experimental procedures were approved by the Shanghai Jiao Tong University School of Medicine Institutional Animal Care & Use Committee (No. A-2015-044).

Control: No treatment.

VCM: VCM was administered by intraperitoneal (i.p.) injection at a dose of 400 mg/kg once a day for 7 days.

VCM+vitamin C (Vit C): vitamin C was administered by i.p. injection at a dose of 200 mg/kg once a day for 8 days starting 1 day before VCM treatment. VCM treatment was the same as in the VCM group.

VCM+NAC: NAC was administered by i.p. injection at doses of 10 mg/kg or 30 mg/kg once a day for 8 days starting 1 day before VCM treatment. VCM treatment was the same as in the VCM group.

Vitamin C: vitamin C was administered by i.p. injection at a dose of 200 mg/kg once a day for 8 days.

NAC: NAC was administered by i.p. injection at a dose of 10 or 30 mg/kg once a day.

Rats in different groups were sacrificed by anesthetic overdose with phenytoin sodium (30 mg/kg) *via* i.p. injection. Plasma separated from blood samples collected from the inferior vena cava was used for blood creatinine (Cre) and urea nitrogen (BUN) detection. One kidney was processed for histopathological evaluation, immunofluorescence staining, or immunohistochemical staining and fixed in 10% paraformaldehyde solution. The other kidney was immersed in physiological saline at 1:9 for glutathione (GSH), superoxide dismutase (SOD), catalase (CAT) and malonaldehyde (MDA) analyses.

### Cre and BUN levels

The levels of Cre and BUN in the serum were detected using commercial assay kits (Nanjing Jiancheng Bioengineering Institute, Nanjing, China) according to the manufacturer's instructions.

### GSH, SOD, CAT activities and MDA contents

GSH, SOD, and CAT activities and MDA contents were detected using specific assay kits according to the manufacturer's instructions (Nanjing Jiancheng Bioengineering Institute, Nanjing, China). In brief, kidney tissues from different groups were processed with physiological saline at 1:9. Lysates were collected to detect GSH, SOD, and CAT activities and MDA contents. Protein concentrations were quantified using the Bradford quantitative protein kit (Bio-Rad).

### Histopathological evaluation, immunofluorescence staining, or immunohistochemical staining

The kidneys of different groups were processed by dehydration in alcohol and embedded in paraffin. Renal sections were cut at a thickness of approximately 5 μm, stained with hematoxylin and eosin (HE), and analyzed under a microscope. Images were captured with a 20× objective lens. For immunofluorescence staining, sections were incubated with caspase-3 antibody (1:100; Cell Signaling Technology, USA) at 4 ℃ overnight and then with fluorescently labeled secondary antibodies at room temperature for 2 hours in the dark. Finally, DAPI was added to the samples to stain the nuclei. Protein was observed under a laser confocal microscope. For immunohistochemistry staining, sections were incubated with KIM-1 (1:100; Absin, Shanghai, China) or NGAL (1:100; Affinity Biosciences, USA) antibody, followed by incubation with biotinylated goat anti-rabbit IgG and the ABC complex. Photographs were taken under a microscope with a 20× objective lens.

### Statistical Analysis

All data is presented as mean ± SEM. Prism version 8.0 (GraphPad software, San Diego, CA, USA) was used for data analysis. Data among multiple groups were analyzed with one-way analysis of variance (ANOVA) followed by Tukey's multiple comparisons test. *p*<0.05 was considered statistically significant.

## Results

### Effect of NAC on the decrease in HK-2 cell viability induced by VCM

**Figure 1:**
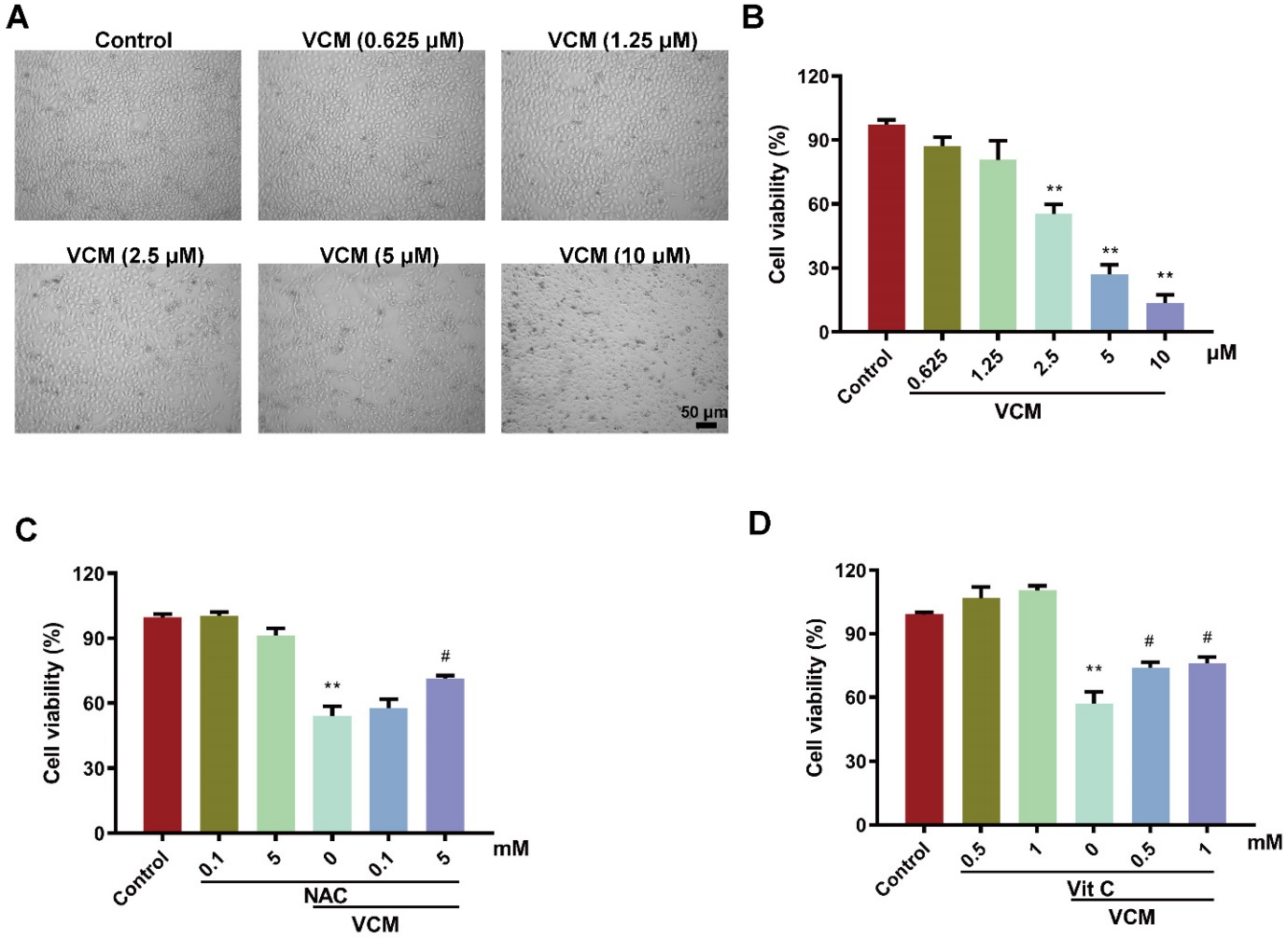
** Effect of NAC on decreased HK-2 cell viability induced by VCM**. (A) Morphological changes in HK-2 cells exposed to VCM. (B) HK-2 cell viability after exposure to different concentrations of VCM detected by CCK-8 assay. (C, D) The effects of NAC and vitamin C on the viability of HK-2 cells exposed to VCM detected by CCK-8 assay. Data was performed from three independent experiments and presented as mean ± SEM. ^**^p<0.01, significantly lower than that in the control group. ^#^*p*<0.05, significantly higher than that in the VCM group. Significance was analyzed by ANOVA followed by Tukey's test.

HK-2 cells were exposed to different concentrations of VCM (0.625-10 μM) for 24 h. Cell viability in the VCM group decreased significantly in a dose-dependent manner compared with that in the control group. HK-2 cells exposed to 2.5 mM VCM exhibited significantly decreased viability and obvious morphological changes compared with control group cells (Figure 1A, B). However, the effect of VCM on cell viability was significantly improved by treatment with NAC (5 mM, Figure 1C) or vitamin C (0.5 mM or 1 mM, Figure 1D). These findings demonstrated that NAC, similar to vitamin C, a highly effective antioxidant, could reverse the decrease in HK-2 cell viability induced by VCM exposure.

### Effect of NAC on metabolic and kidney function changes induced by VCM in rats

No significant difference was observed in the initial body weight among the eight experimental groups of animals. However, body weight and food intake decreased after VCM treatment for 7 days compared with those in the control group. The kidney weight and kidney index in the VCM group were significantly increased compared with those in the control group. Treatment with NAC (10, 30 mg/kg) or vitamin C (200 mg/kg) effectively prevented VCM-induced changes in body weight, food intake, kidney weight and the kidney index. Interestingly, the administration of NAC alone did not affect the above metabolic parameters. All metabolic parameters are shown in Table 1.

**Figure 2:**
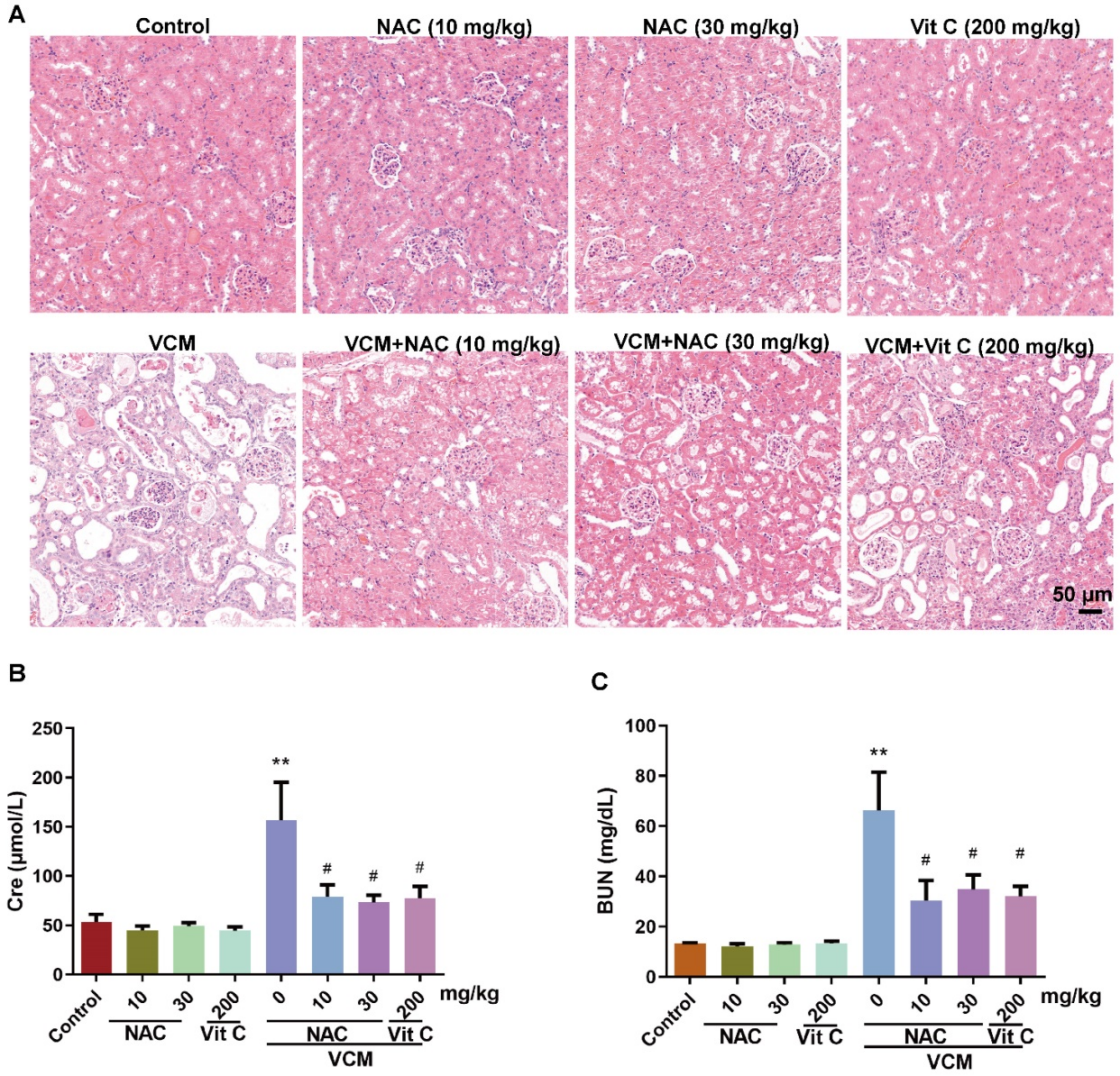
** Effect of NAC on changes in renal histopathology and kidney function induced by VCM in rats.** (A) Sections of kidneys from different groups stained using HE. (B, C) Serum levels of Cre and BUN. Data was presented as mean±SEM (n=5 rats). ^**^p<0.01, significantly higher than that in the control group. ^#^*p*<0.05, significantly lower than that in the VCM group. Significance was analyzed by ANOVA followed by Tukey's test.

HE-stained kidney samples showed that (Figure 2A) treatment with VCM for 7 days induced extensive renal tissue damage, such as tubular degeneration, atrophy, dilation, necrosis, cast formation, interstitial edema and inflammatory cell infiltration, compared with the control condition. VCM-induced kidney injury could be reversed by NAC or vitamin C, while NAC or vitamin C administration alone did not affect these pathologic changes. In addition, VCM-induced kidney dysfunction caused by increased serum Cre and BUN levels was also significantly reversed by NAC or vitamin C treatment (Figure 2B, 2C). These results demonstrated that NAC can improve VCM-induced changes in metabolic parameters and kidney function.

### Effect of NAC on VCM-induced increased expression of KIM-1 and NGAL in HK-2 cells and rat kidneys

**Figure 3:**
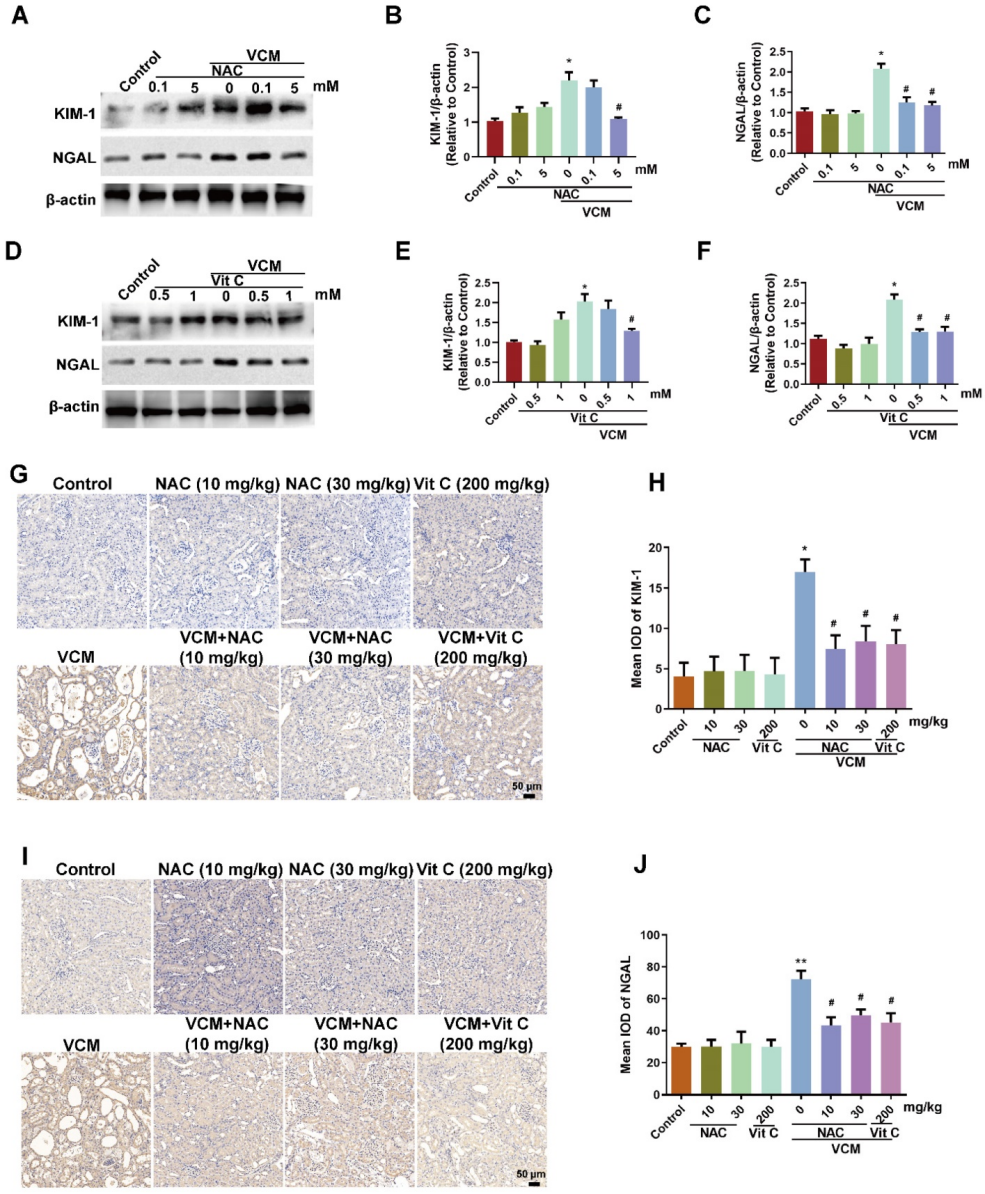
** Effect of NAC on VCM-induced increased expression of KIM-1 and NGAL in HK-2 cells and rat kidneys**. The effects of NAC (A, B, C) and vitamin C (D, E, F) on the expression of KIM-1 and NGAL in HK-2 cells using western blot and semiquantified analysis. (G, H) KIM-1 expression and analysis in kidney tissue detected using immunohistochemistry. (I, J) NGAL expression and analysis in kidney tissue detected using an immunohistochemical technique. Data was performed from three independent experiments or 5 rats and presented as mean ± SEM. ^*^p<0.05, significantly higher than that in the control group. ^#^*p*<0.05, significantly lower than that in the VCM group. Significance was analyzed by ANOVA followed by Tukey's test.

KIM-1 and NGAL are considered as the most promising biomarkers in renal tubular injury. After VCM treatment for 24 h, KIM-1 and NGAL were increased significantly in HK-2 cells, and the increased KIM-1 and NGAL expression was reversed by NAC (5 mM) or vitamin C (0.5 mM, 1 mM) (Figure 3A, B, C, D, E and F). Similar results were also found in VCM-treated rat kidneys, while VCM-induced nephrotoxicity causing decreased KIM-1 and NGAL expression could be reversed by NAC or vitamin C treatment (Figure 3G, H, I and J). These results demonstrated that NAC could confer effective protection against VCM-induced renal injury by decreasing KIM-1 and NGAL expression.

### Effect of NAC on VCM-induced cell apoptosis both *in vitro* and *in vivo*

**Figure 4:**
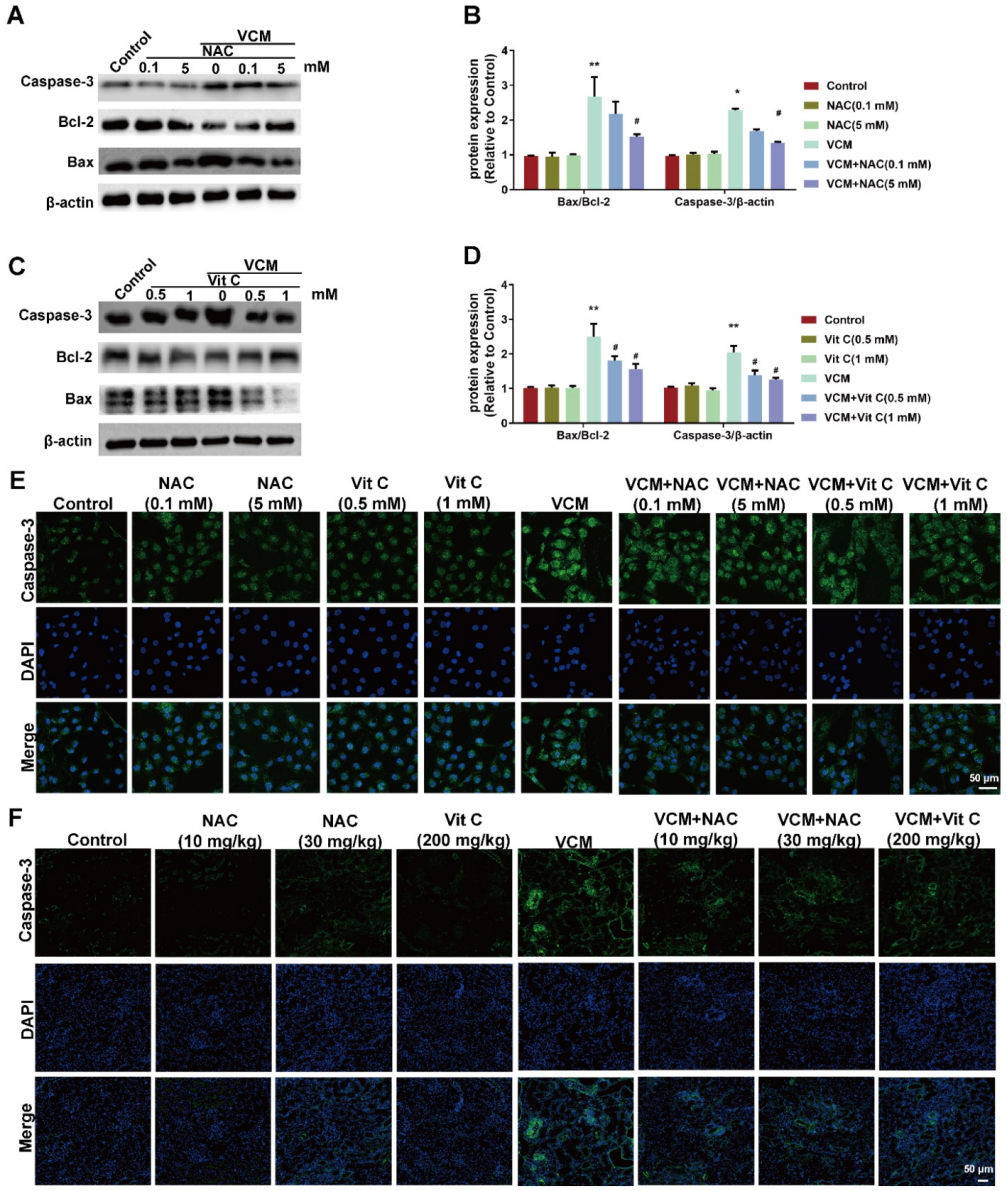
** Effect of NAC on VCM-induced cell apoptosis in HK-2 cells and rat kidneys. (**A, B, C, D) The expression and analysis of Bax, Bcl-2 and caspase-3 detected and analyzed by western blot in HK-2 cells treated with VCM and NAC or vitamin C. (E) Caspase-3 expression and analysis using immunofluorescence staining in HK-2 cells treated with VCM and NAC or vitamin C. (F) Caspase-3 expression and analysis using immunofluorescence staining in rat kidneys treated with VCM and NAC or vitamin C. Data was performed from three independent experiments or 5 rats and presented as mean ± SEM. ^*^p<0.05, ^**^p<0.01, significantly higher than that in the control group. ^#^*p*<0.05, significantly lower than that in the VCM group. Significance was analyzed by ANOVA followed by Tukey's test.

Cell apoptosis was observed in VCM-treated HK-2 cells and kidney tissue, as shown in Figure 4. Upregulated Bax and caspase-3 expression and downregulated Bcl-2 expression were observed in VCM-treated HK-2 cells (Figure 4A, B, C, D and E), and upregulated expression of caspase-3 was also observed in VCM-treated kidneys compared to control group (Figure 4F). NAC and vitamin C prevented the VCM-induced changes described above by increasing Bcl-2 expression and decreasing Bax and caspase-3 expression. In addition, apoptosis in HK-2 cells or rat kidneys was not affected by NAC or vitamin C treatment alone. These results indicated that NAC could prevent or attenuate cell apoptosis induced by VCM in the *in vitro* and* in vivo* experiments.

### Effect of NAC on oxidative stress induced by VCM in HK-2 cells and kidney tissue

**Figure 5:**
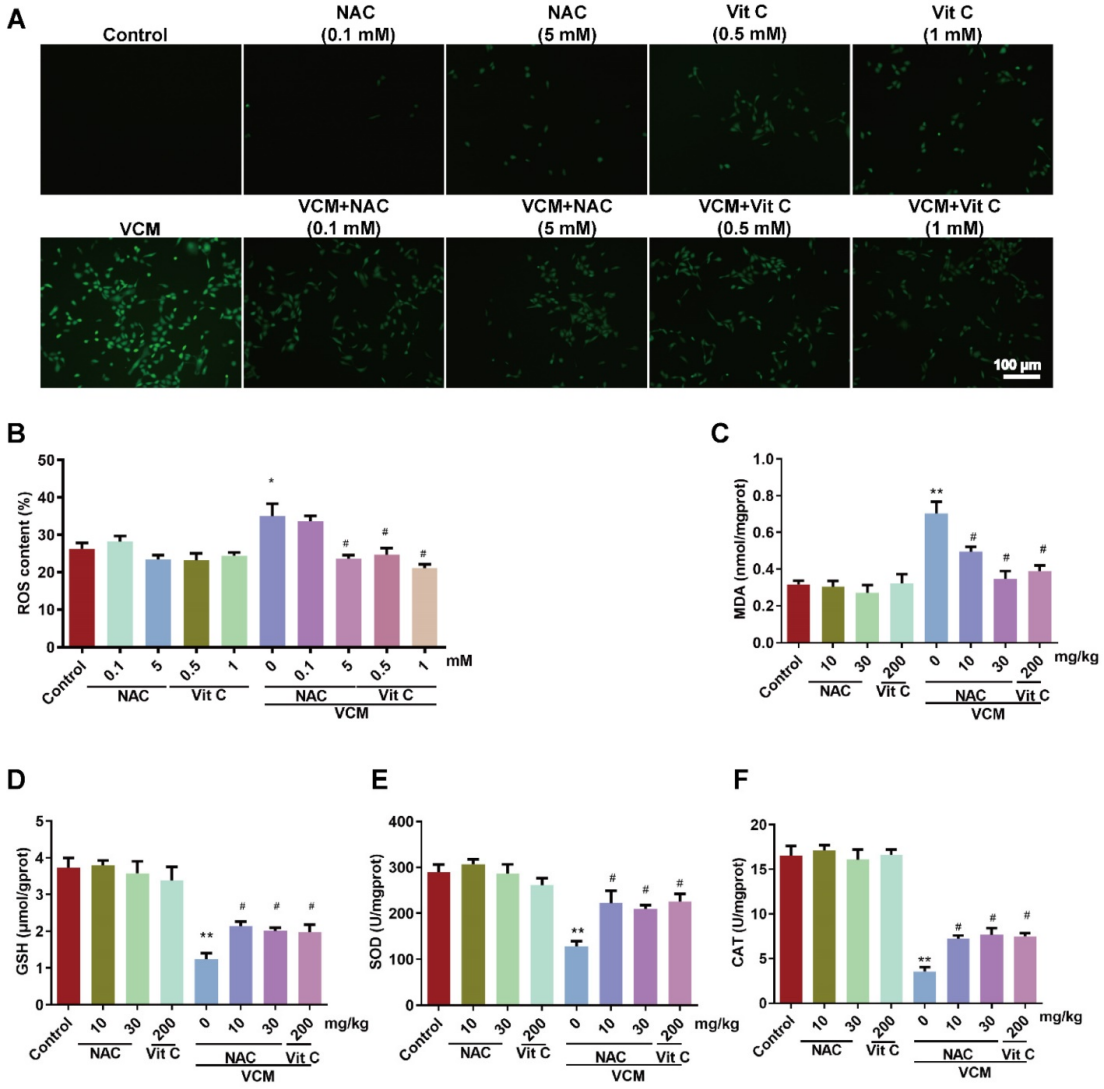
** Effect of NAC on oxidative stress induced by VCM in HK-2 cells and rat kidneys.** (A) ROS production in HK-2 cells detected by immunofluorescence. (B) Quantified analysis of ROS levels in HK-2 cells. (C) MDA contents in rat kidneys from different groups. (D) GSH contents in rat kidneys. (E) SOD activity in rat renal tissue. (F) CAT activity in rat renal tissue. Data was performed from three independent experiments or 5 rats and presented as mean ± SEM. ^*^p<0.05, ^**^p<0.01, significantly different compared with that in the control group. *^#^p*<0.05, significantly different compared with that in the VCM group. Significance was analyzed by ANOVA followed by Tukey's test.

Increased ROS production (Figure 5A, 5B) was observed in HK-2 cells exposed to VCM for 6 h. Interestingly, HK-2 cells incubated with NAC or vitamin C and VCM showed a lower ROS intensity than cells treated with VCM alone. Furthermore, increases in MDA (Figure 5C) and decreases in GSH (Figure 5D), SOD (Figure 5E) and CAT activities (Figure 5F) induced by VCM treatment were also observed in rat kidneys. These events during oxidant injury were significantly reversed by NAC or vitamin C. These results demonstrated that NAC, as an antioxidant, could protect the kidney from the damage caused by VCM by decreasing oxidative damage.

### Effect of NAC on the VCM-activated P38 MAPK/JNK signaling pathway in HK-2 cells

**Figure 6:**
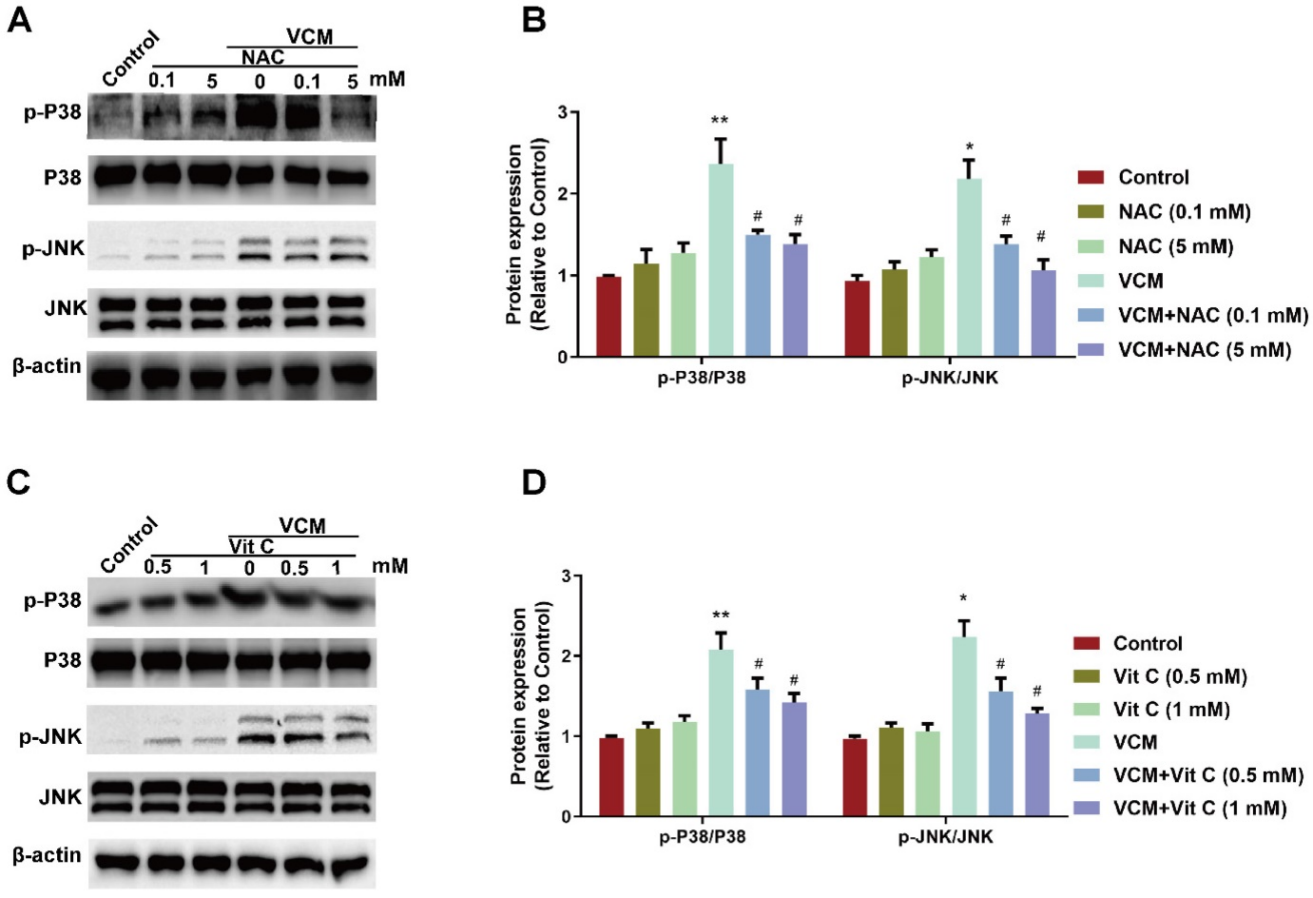
** Effect of NAC on the VCM-activated P38 MAPK/JNK signaling pathway**. (A) Phosphorylation levels of P38 MAPK and JNK in HK-2 cells treated with VCM and NAC. (B) Phosphorylation levels of P38 MAPK and JNK in HK-2 cells treated with VCM and vitamin C. Data was performed from three independent experiments and presented as mean ± SEM. ^*^p<0.05, ^**^p<0.01, significantly higher than that in the control group. ^#^*p*<0.05, significantly lower than that in the VCM group. Significance was analyzed by ANOVA followed by Tukey's test.

Evidence has shown that oxidative stress affects MAPKs, which function in intracellular signaling pathways involved in the process of VCM-induced renal cell apoptosis. Thus, abnormal changes in the P38 MAPK/JNK signaling pathway along with renal oxidative damage were also found in our results (Figure 6). Increased phosphorylation of JNK and P38 MAPK were detected in HK-2 cells during VCM treatment. Treatment with NAC (Figure 6A and B) or vitamin C (Figure 6C and D) effectively regulated the P38 MAPK/JNK signaling pathway by decreasing P38 MAPK and JNK phosphorylation. In addition, the signaling pathway was not regulated by NAC or vitamin C treatment alone compared with the control condition. These results demonstrated that the P38 MAPK/JNK signaling pathway activated by VCM was involved in the protective effects of NAC.

## Discussion

The study results are as follows. Firstly, the results demonstrated that VCM-induced acute kidney injury with increased BUN and Cre and histological kidney damage could be prevented or ameliorated by NAC. Second, the increased expression of caspase-3, KIM-1 and NGAL induced by VCM was also reversed by NAC in the *in vivo* and *in vitro* experiments. Third, NAC could significantly improve VCM-induced renal injury by inhibiting ROS production, decreasing cell apoptosis associated with a decreased Bax/Bcl-2 ratio and caspase-3 expression in HK-2 cells, and attenuating oxidative kidney damage by decreasing GSH, SOD, and CAT activities and increasing MDA levels. Finally, the results also demonstrated that the P38 MAPK/JNK signaling pathway was involved in the protective effect of NAC against VCM-induced renal oxidative damage. The above results indicated that NAC played an important role in the protective effect against VCM-induced nephrotoxicity, and the protective effects were at least partly related to regulation of oxidative stress and apoptosis *via* the P38 MAPK/JNK signaling pathway.

VCM is one of the most frequently employed clinical therapies for MRSA infections 20. However, nephrotoxic effects as a major concern during VCM therapy are closely associated with high doses or prolonged durations of VCM administration, thus, with limiting clinical applications 21. Nephrotoxicity has been reported an occurrence in 5%-35% of patients who accepted VCM therapy 22. Evidence shows that VCM-induced nephrotoxicity correlates with oxidative stress because antioxidants can ameliorate some adverse effects of VCM therapy 23, 24. Histopathological damage in VCM-treated rat kidneys is characterized by tubular degeneration, atrophy, dilation, necrosis, cast formation and interstitial edema 25. Serum BUN and Cre levels are important biochemical parameters of renal dysfunction that are also significantly increased with VCM therapy 26. In this research, renal histopathological damage, as well as renal dysfunction associated with increased Cre and BUN levels, was obvious after 400-mg VCM treatment once daily for 7 days. Along with VCM-induced renal histopathological damage and dysfunction, metabolic parameter changes, including decreased food intake and body weights and increased kidney weights and ratios, were also observed. These renal pathological events during VCM treatment were consistent with those observed in previously reported studies 8. Interestingly, in our present study, we found that these renal pathological injuries could be attenuated by the antioxidants NAC and vitamin C. As a powerful antioxidant, vitamin C has been reported to be applied in clinical therapy for VCM-induced nephrotoxicity 5, 27 and was selected as the positive control drug in this study. However, high-dose vitamin C ingestion was required due to vitamin C's poor stability and easy degradation in aqueous medium, which might result in nephrotoxicity 28, 29. NAC has strong antioxidant potential as a powerful treatment agent, attenuating oxidative stress-induced cell injury and other pathological events, and is most frequently used in clinical and experimental research despite its classic use as a mucoactive agent 30. Cela et al. have summarized the nephroprotective effects of NAC in their work and pointed out that NAC could be used as an adjuvant treatment ingredient in urinary tract infections to reduce antibiotic drugs nephrotoxic adverse reactions 31, 32. These results provided more support that NAC has great clinical value as a renal protective agent of vancomycin.

In this study, we also found that KIM-1 and NGAL expression was significantly enhanced after kidney injury. KIM-1 is a transmembrane glycoprotein located in renal proximal convoluted tubule epithelial cells 33, and NGAL is expressed in the tubular epithelium and upregulated in cells damaged due to kidney injury 34. In the process of renal dysfunction induced by gentamicin or diabetes mellitus, KIM-1 and NGAL were reported to be significantly increased due to oxidative stress, which was proposed as the main cause, and antioxidants could effectively inhibit KIM-1 and NGAL expression 35, 36. Previous studies have reported that KIM-1 and NGAL are sensitive biomarkers for kidney histopathologic damage caused by VCM 37, 38. In this study, upregulated expression of KIM-1 and NGAL in rat kidneys was also detected in the presence of kidney damage from VCM, which could be attenuated by the antioxidants NAC and vitamin C, suggesting that an antioxidant effect is involved in the protective effect of NAC against VCM-induced renal injury by decreasing KIM-1 and NGAL expression. In addition, VCM-induced kidney injury was ameliorated by NAC, which attenuated renal pathological injury and kidney dysfunction, enhanced renal tubular cell survival, improved metabolic parameters, and exhibited antioxidant and antiapoptotic properties.

In fact, the mechanism of VCM-induced renal damage is still unclear, and oxidative stress is considered a major cause of kidney injury. Thus, a large number of antioxidants, including vitamin E, vitamin C and NAC, can prevent such damage probably via antioxidative stress effects 11, 39, 40. NAC showed a significant ameliorating effect on lead-induced ROS, DNA damage, and apoptosis. VCM treatment could stimulate free radical production, resulting in cellular injury through various mechanisms, including DNA damage, lipid peroxidation and protein denaturation. These changes cause damage to the membrane structure and function with increased MDA levels, which are regarded as indicators of lipid peroxidation 41. The protective effect against oxidative stress, including the antioxidative enzymes SOD, CAT and GSH, is widely accepted as a powerful defense mechanism against oxidative kidney damage 42. During VCM-induced kidney injury, all defense enzyme activities reportedly declined 8, 25, which is consistent with our results. VCM treatment enhanced ROS production in HK-2 cells and induced oxidative metabolism with increased MDA levels and decreased GSH, SOD and CAT activities in the rat kidneys, indicating that VCM resulted in an oxidative status in the process of renal damage. The antioxidant NAC reversed abnormal changes in oxidative stress indicators and downstream histopathological damage by augmenting the activities of GSH, SOD and CAT and decreasing the level of MDA in VCM-damaged kidneys. ROS production in VCM-treated HK-2 cells was also inhibited by NAC. The protective function of NAC against VCM-induced renal oxidative stress may be related to the underlying mechanism of its antioxidant properties reported in numerous studies 43. An opposing perspective has also been reported in other studies, where NAC at 100 or 300 μM and vitamin C at 30 or 100 μM did not show significant protection against VCM-induced renal tubular cell injury 44. We did not observe the exact treatment condition of NAC or vitamin C and VCM in the kidney cells. In our study, we also found no protective effect at lower concentrations of NAC (0.1 mM) or vitamin C (<0.5 mM) or with simultaneous treatment with VCM (data not shown). However, pretreatment with NAC (5 mM) or vitamin C (0.5, 1 mM) for 2 h resulted in significant protection against VCM in HK-2 cells. We have reasons to believe that the high doses and preconditioning systems in our study may have supplied an antioxidative atmosphere in advance, which is required to produce a significant protective effect on reducing VCM-associated nephrotoxicity, which is consistent with previous *in vivo* studies 27.

Oxidative stress has been implicated as a potential mechanism responsible for the pathogenesis of VCM-induced nephrotoxicity. The oxidative stress reaction leads to expression of the apoptosis-related genes Bax and Bcl-2, which act on the mitochondrial membrane to modulate mitochondrial permeability and cytochrome C release as proapoptotic and antiapoptotic proteins, respectively, play an important role in regulating apoptosis, while an increased Bax and Bcl-2 ratio may directly correspond to apoptosis 45, 46. Caspase activation, a common event in apoptosis via the extrinsic and intrinsic pathways, has been reported in VCM-induced apoptosis 8. VCM-induced mitochondrial membrane depolarization and cytochrome C release resulted in caspase-3 activation during apoptosis in damaged renal tubular cells 47. Our present results demonstrated that VCM-induced renal damage was accompanied by increased caspase-3 activity, Bax/Bcl-2 ratio. However, renal caspase-3 and Bax expression was decreased and Bcl-2 expression was increased with NAC and vitamin C treatment. Our findings indicated that the protective effect of NAC on attenuating VCM-induced nephrotoxicity was mainly mediated by antioxidant and antiapoptotic properties.

VCM-induced nephrotoxicity is a complex process involving many signaling pathways. Oxidative stress can affect intracellular signaling pathways, such as those involving MAPKs which may play an important role in VCM-induced acute kidney injury 8. MAPK signaling pathways are well studied and involved in various cellular processes, including cell inflammation, apoptosis, differentiation and proliferation 48, 49, which are also evident in kidney injury 50. The P38 MAPK/JNK pathways can modulate the expression of the apoptotic proteins Bax and Bcl-2, which is mediated by oxidative stress-induced renal damage 47, 51, 52. In the present study, we found that kidney injury is related to VCM-induced oxidative stress, apoptosis and the expression of the apoptosis-related genes Bax and Bcl-2, as well as P38 MAPK/JNK phosphorylation were found to be significantly upregulated but were decreased by the antioxidants NAC and vitamin C. Our results indicated that the P38 MAPK/JNK pathways are involved in the protective effect of NAC against VCM-induced nephrotoxicity.

The process of kidney injury involves complex signal networks and the precise mechanism is still unclear. In this study, we have confirmed that vancomycin-induced renal injury resulted in abnormal levels of mitochondria-related oxidative stress. Oxidative stress and autophagy interacted in different pathological conditions 53. A large amount of evidence indicated that reactive oxygen species acted as upstream regulators to induce the autophagy 54, 55. Autophagy action was reported to produce harmful effects on renal injury under pathological conditions 56, 57. Vancomycin-induced renal injury could be significantly reversed by autophagy intervention 58. As a classic antioxidant, NAC could stabilize mitochondrial function and might play a protection in kidney via autophagy related mechanism. However, more explorations are needed which will be performed in our further study.

In summary, our results demonstrated that NAC has a protective effect against nephrotoxicity from VCM therapy by inhibiting oxidative stress and apoptosis *via* the P38 MAPK/JNK signaling pathway, and we believe that supplementation with antioxidants such as NAC provides a valuable therapeutic benefit in the clinical application of VCM.

## Figures and Tables

**Figure 7:**
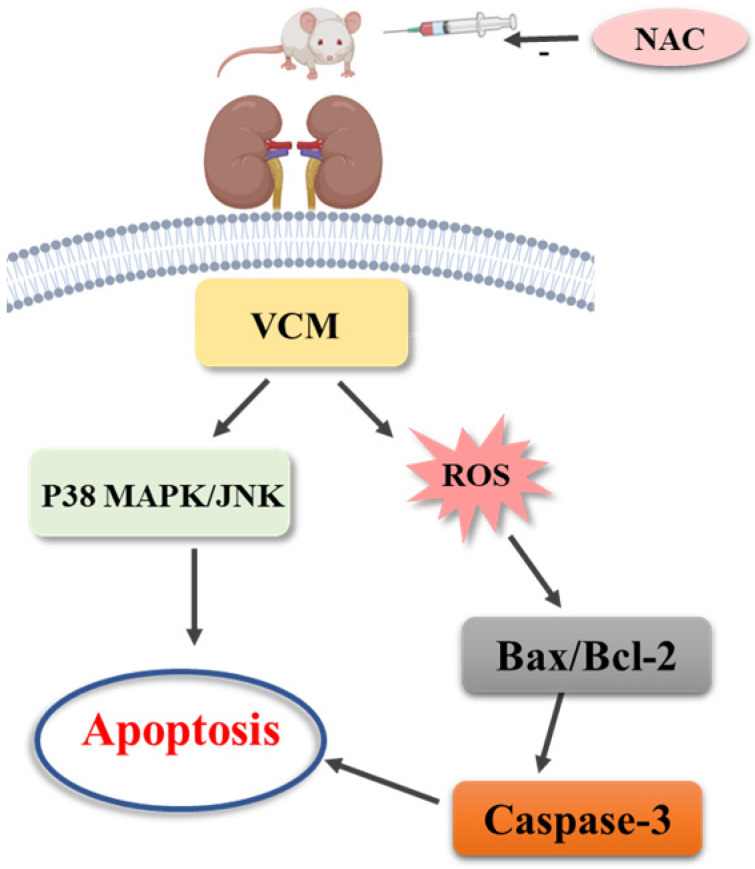
Mechanism of N-acetylcysteine against VCM-induced nephrotoxicity.

**Table 1:** Influence of NAC in VCM-induced changes of metabolic parameters.

	Control	NAC (10 mg/kg)	NAC (30 mg/kg)	Vit C (200 mg/kg)
Initial weight (g)	202.68±4.74	204.90±3.15	206.82±4.07	201.94±3.73
Final weight (g)	258.64±9.83	235.83±7.95	231.71±14.90	249.89±8.16
Kidney weight (g)	1.01±0.05	0.89±0.04	0.96±0.06	0.90±0.03
Kidney index (%)	0.40±0.02	0.36±0.03	0.41±0.02	0.47±0.05
Food intake (g)	20.72±0.96	19.29±1.91	19.97±4.80	18.31±2.25
	VCM	VCM+NAC (10 mg/kg)	VCM+NAC (30 mg/kg)	VCM+Vit C (200 mg/kg)
Initial weight (g)	210.33±5.50	209.16±8.43	206.28±1.73	204.65±4.65
Final weight (g)	179.27±7.98^**^	230.53±10.70^#^	206.92±11.29^#^	204.17±9.05^#^
Kidney weight (g)	2.48±0.96^**^	1.16±0.08^#^	1.28±0.13^#^	1.19±0.09^#^
Kidney index (%)	1.36±0.41^**^	0.50±0.04^#^	0.59±0.07^#^	0.58±0.03^#^
Food intake (g)	5.18±1.32^**^	14.28±1.32^#^	10.88±1.80^#^	10.79±2.08^#^

**Table 1.** Data was performed from 5 rats and presented as mean ± SEM. ^**^p<0.01, significantly different with that in the control group. ^#^*p*<0.05, significantly different with that in the VCM group. The significance was analyzed by ANOVA followed by Tukey's test.
